# Endoscopic thoracic sympathectomy for hyperhidrosis: Technique and results

**DOI:** 10.4103/0972-9941.38907

**Published:** 2007

**Authors:** C S Cinà, M M Cinà, C M Clase

**Affiliations:** Division of Vascular Surgery, Department of Surgery, University of Toronto and Epidemiology and Biostatistics, McMaster University, Hamilton, Canada; 1 Student Toronto French School, Canada; 2 Division of Nephrology, Department of Medicine and Epidemiology and Biostatistics, McMaster University, Hamilton, Canada

**Keywords:** Endoscopic sympathectomy, hyperhidrosis, quality of life

## Abstract

**Outline:**

We review the clinical features of hyperhidrosis and the range of treatments used for this condition. We describe in detail the technique of endoscopic sympathectomy. We summarize studies that have reported results of endoscopic sympathectomy. We present new data highlighting the difference in quality of life between patients with hyperhidrosis and controls.

## INTRODUCTION

Hyperhidrosis is a dysregulation of the neural sympathetic control of the eccrine sweat glands, which leads to excessive and unpleasant sweating. The thoracic sympathetic chain is involved in the neural control of sweating in hyperhidrosis of the upper limbs. It lies in front of the neck of the thoracic ribs and under the parietal pleura. Preganglionic sympathetic fibers (white rami) synapse with ganglionic cells of the sympathetic chain, usually at the same level of the lateral horn of the spinal cord where they originate. Occasionally, these preganglionic fibers travel along the sympathetic chain downward or more commonly upward before joining a ganglionic cell at a different level. The postganglionic fibres (gray rami) join a peripheral nerve or distribute around a segmental artery to reach the effector cell. The nerve endings, which control the function of the glands involved in hyperhidrosis, differ from other sympathetic fibers in that they use acetylcholine as the neurotransmitter, which is why anticholinergic drugs are sometimes used in the treatment of this condition. Symptoms affect important aspects of quality of life including physical, psychological and social dimensions.

Excessive sweating usually starts in childhood or adolescence and continues throughout life. Nervousness and anxiety can cause or aggravate sweating. Sweating can also be triggered by high environmental temperatures and emotional stress or it may appear without any obvious reasons. Usually, hyperhidrosis worsens during the warmer seasons and improves during the winter. People who suffer hyperhidrosis can experience excessive sweating on their hands (palmar), on their armpits (axillary), on their feet (plantar), on their face (facial) or on their trunk (truncal). Many individuals suffer from a combination of the above. The prevalence and incidence of the condition in North America are unknown. In a population-based study in Israel the prevalence in young adults was 0.6–1.0%.[1] Men and women appear to be equally likely to be affected. Although this condition appears to have a large impact on the quality of life, the true magnitude is difficult to quantify. Several scales have been designed or used: generic tools such as the SF36[2–4] and specific measures such as the Dermatology Life Quality Index (DLQI)[5] and Keller[6] and Milanez de Campo[7] questionnaires. We have validated and used the Illness Intrusiveness Rating Scale and ten disease-specific severity questions in this setting.[3]

## TREATMENTS

There are several ways to treat excessive sweating: Antiperspirants (e.g. Aluminium Zirconium, Tetrachlorohydrex Glycine), iontophoresis, anticholinergic medication, psychotherapy, botulinum toxin injections and surgery. Recently, there is increased awareness with the use of botulinum toxin injections as a non-surgical treatment of hyperhidrosis. Although indications have been made that botulinum toxin is effective in reducing sweating, the results are generally not durable beyond 14 months.[8] Also, the side effects of this treatment can involve temporary muscle weakness or partial paralysis[9,10] and the injection procedure is technically demanding, involving up to 35 injection sites in each palm.[9] For patients whose disease is sufficiently severe that they would consider botulinum toxin injections, we believe that endoscopic thoracic sympathectomy (ETS) offers a safe and durable alternative. The latter requires excision, electrocoagulation of or application of surgical clips[11] on the thoracic sympathetic ganglia between T2 and T4 or T5. In the past, this surgery required bilateral thoracotomies and therefore it was rarely offered. ETS is a minimally invasive technique that has increased the interest in this modality of treatment.

## ENDOSCOPIC SYMPATHECTOMY: SURGICAL TECHNIQUE

**Figure 1:**
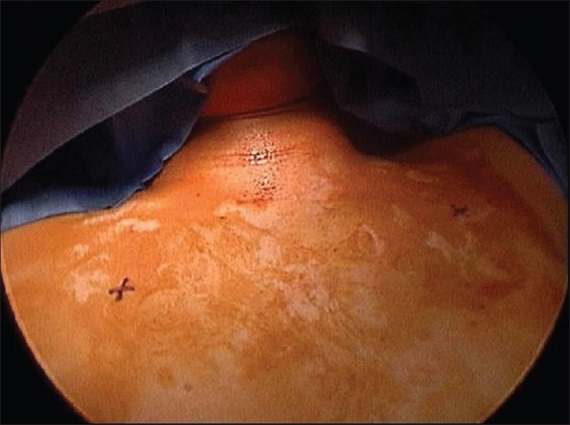
Position of the patient and surgical field. The skin marks refer to the thoracoscopic ports entry sites

**Figure 2:**
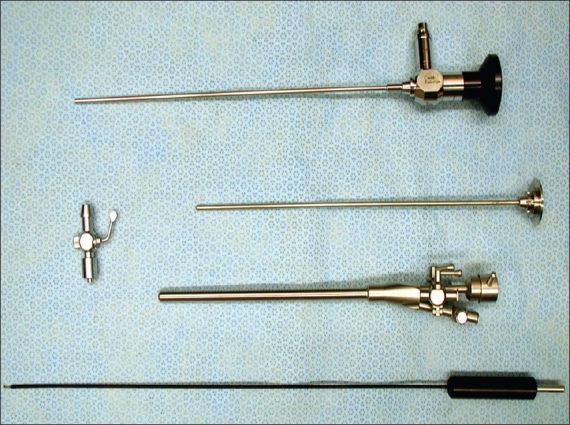
Custom made operating thoracoscope for endoscopic thoracic sympathectomy

**Figure 3:**
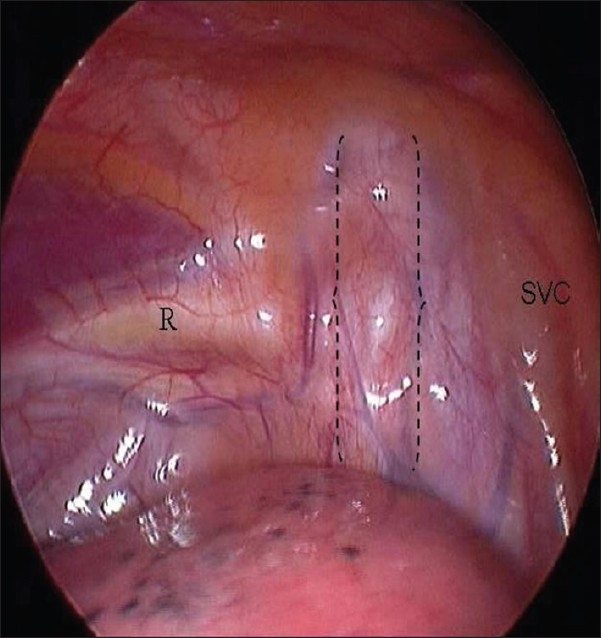
Endoscopic view of the right hemithorax. The curly brackets delimitate the sympathetic chain. R = second rib; SVC = superior vena cava

**Figure 4:**
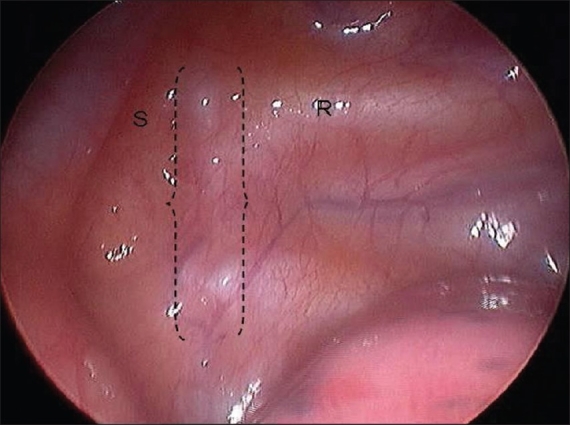
Endoscopic view of the left hemithorax. The curly brackets delimitate the sympathetic chain. R = second rib; S = subclavian artery

We perform bilateral ETS under general anesthesia using a single lumen endotracheal tube. Patients are positioned supine with both arms outstretched on arm boards and the trunk in a 30° Fowler position. The sterile field includes the neck, both axillae and upper arms down to the costal margin bilaterally [Figure 1]. A specifically designed and custom made 5 mm rigid scope is used [Figure 2]. This has a zero degree angle lens, an aspirating channel and an operating channel. The thoracoscope is introduced through a port placed in the mid-clavicular line in the second intercostal space. CO2 is insufflated to 8–10 cmH2O of pressure to collapse the dome of the lung. The thoracic chain is readily identified covered by the thin layer of the parietal pleura [Figures 3 and 4]. A diathermy hook is inserted through the operating channel of the thoracoscope. The sympathetic chain is visualized behind the parietal pleura, which is then scored on either side using the cautery to delineate the position of the chain and the extent of the planned cauterization which corresponds to the extent of the chain destroyed [Figures 5 and 6]. Using the ribs for reference, the sympathetic chain is then cauterized and divided from T2 to T3 for patients with predominantly palmar hyperhidrosis and from T2 to T4 for patients with predominantly axillary hyperhidrosis. The thoracoscope is then removed and replaced with a small red rubber catheter. With positive pressure ventilation, the catheter is then removed under suction to allow expansion of the lung. The skin incision is closed with a single absorbable 3–0 suture followed by placement of skin tapes. Contralateral sympathectomy is performed in a similar manner without changing the patient's position.

**Figure 5A:**
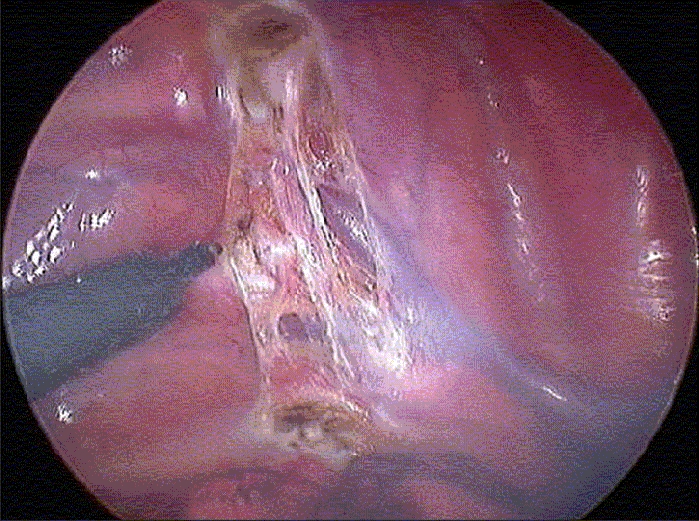
The parietal pleura has been scored with diathermy on either side of the chain, and the upper and lower border of the chain have been coagulated. A - Right hemithorax; B Left hemithorax

**Figure 5B:**
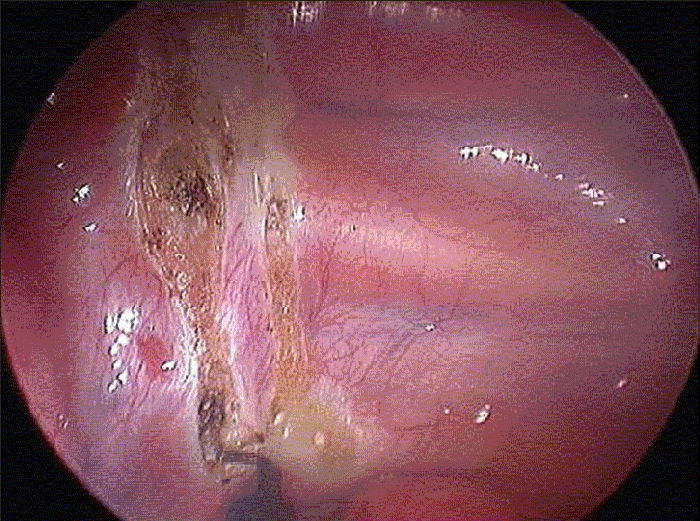
The parietal pleura has been scored with diathermy on either side of the chain, and the upper and lower border of the chain have been coagulated. A - Right hemithorax; B Left hemithorax

**Figure 6:**
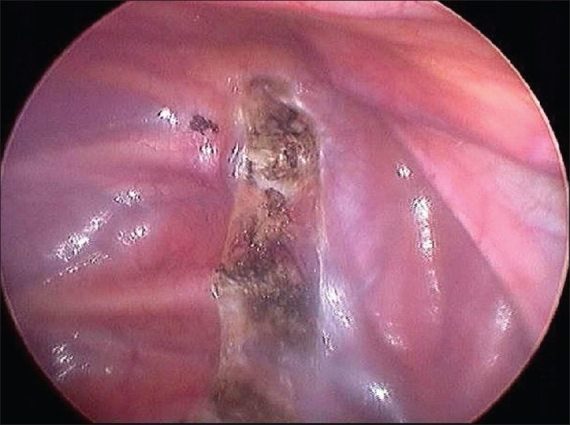
The right sympathetic chain has been coagulated from T2 to T4

## QUALITY OF LIFE IN PEOPLE WITH HYPERHIDROSIS

In clinical practice and for research, we found the Illness Intrusiveness Rating Scale [Table 1] very useful in the assessment of the impact of hyperhidrosis on patients' lives and of the overall response to surgery in patients who undergo ETS. Respondents use a seven-point Likert scale to assess the impact of their disease on each of 13 domains: health, diet, work, active and passive recreation, financial situation, relationship with spouse, sex life, family and other social relations, self expression/self improvement, religious expression and community/civic involvement. The minimum score is 13 and the maximum 91. The scale has been validated and used in patients with a number of chronic diseases. [12–16] In patients with hyperhidrosis we have previously shown that the IIRS is valid by comparing responses on this scale with responses to a global severity item and ten additional severity questions, based on theoretical constructs, clinical observations and contributions from hyperhidrosis patients.[3] Internal consistency (Cronbach's α = 0.88) and test-retest reliability (intraclass correlation coefficient = 0.89) were shown to be very good in patients with hyperhidrosis.[3] We also use the ten severity questions described above [Table 2].[3]

**Table 1:** Questions of the Illness Intrusiveness Rating Scale

Health:
Not Very Much 1 2 3 4 5 6 7 Very Much
Diet (i.e., the things you eat and drink):
Not Very Much 1 2 3 4 5 6 7 Very Much
Work:
Not Very Much 1 2 3 4 5 6 7 Very Much
Active recreation (e.g., sports):
Not Very Much 1 2 3 4 5 6 7 Very Much
Passive recreation (e.g, reading, listening to music):
Not Very Much 1 2 3 4 5 6 7 Very Much
Financial situation:
Not Very Much 1 2 3 4 5 6 7 Very Much
Relationship with your spouse (girlfriend or boyfriend if not married):
Not Very Much 1 2 3 4 5 6 7 Very Much
Sex life:
Not Very Much 1 2 3 4 5 6 7 Very Much
Family relations:
Not Very Much 1 2 3 4 5 6 7 Very Much
Other social relations:
Not Very Much 1 2 3 4 5 6 7 Very Much
Self-expression/self-improvement:
Not Very Much 1 2 3 4 5 6 7 Very Much
Religious expression:
Not Very Much 1 2 3 4 5 6 7 Very Much
Community and civic involvement:
Not Very Much 1 2 3 4 5 6 7 Very Much

**Table 2:** Questions designed to explore the severity of sweating

How do you describe your condition? (If you had surgery, please answer describing your present condition and not the one before surgery)
Mild 1 2 3 4 5 6 7 severe
If you had surgery how do you rate the results of this treatment?
No improvement 1 2 3 4 5 6 7 Major improvement
Do you use cosmetic preparations (topical powders, astringents, etc)?
Times in one day 0 1 2 3 4 5 6 ≥7
Do you use medications in pill form (anticholinergic, sedatives)?
Times in one day 0 1 2 3 4 5 6 >7
Do you use any other device or treatment (i.e., iontophoresis or electronic devices) for hyperhidrosis?
Times in one month 0 1 2 3 4 5 6 ≥7
How many times in a day do you shower/bathe because of excessive sweating?
Times in one day 1 2 3 4 5 6 ≥7
How many times in a day do you change your clothes because of excessive sweating?
Times in one day 1 2 3 4 5 6 ≥7
Does excessive sweating limit the kinds of clothes you wear (in terms of colours, material or design)?
Not Very Much 1 2 3 4 5 6 7 Very Much
How much does alcohol increase sweating or blushing?
Not Very Much 1 2 3 4 5 6 7 Very Much
How much does spicy food increase sweating or blushing?
Not Very Much 1 2 3 4 5 6 7 Very Much
How much do caffeine-containing drinks (coffee, chocolate, cola, etc.) increase sweating or blushing?
Not Very Much 1 2 3 4 5 6 7 Very Much

In our study of 30 patients with primary, bilateral hyperhidrosis, the impact of hyperhidrosis on quality of life was demonstrated by the high preoperative IIRS score (57 ± 14) observed in this group of patients referred to vascular surgeons, similar to that seen in patients with severe debilitating chronic conditions, such as rheumatoid arthritis, end-stage renal disease and multiple sclerosis.[13,17] The domains, which appeared most affected (median score 5 or greater) were work, active recreation, relation with spouse, sex life, other social relations, self-expression/self-improvement and community and civic involvement.

Ramos and colleagues have used the State-Trait Anxiety Inventory to document high levels of anxiety in 106 patients with hyperhidrosis.[18,19] Many of these patients also reported symptoms more commonly associated with anxiety: palpitations, trembling, non-specific headache and gastrointestinal disturbance.

In addition, we have recently compared the preoperative quality of life in these patients with hyperhidrosis with that of controls. The control group was a random sample of eleven English and French speaking individuals older than 15 years of age not affected by hyperhidrosis identified among students and teachers in a Toronto French Schools, office assistants and physicians’ offices [Table 3]. Controls and people with hyperhidrosis provided significantly different answers to the questions addressing correlates of severity of disease [Table 4]. Compared with controls, patients with hyperhidrosis more frequently used antiperspirants, took more showers or baths, changed their clothing more often, were limited in the type of wardrobe that they could use and their sweating was made worse by alcohol, spicy foods and caffeine containing drinks. On the IIRS, the control group, as expected, had a total score of 13.5 SD 0.7, while the group of individuals with hyperhidrosis scored 57 SD 14. This difference was highly statistically different (*P* = 0.001). For all questions of the IIRS, the experimental group rated their health problems as more intrusive than the control group [Table 5].

**Table 3:** Demographics in the control and experimental group

	Control group (N=11)	Experimental group (N = 30)	*P*
Age, mean ± SD	39 ± 13	26 ± 10	0.05
Male sex, %	36	50	ns
Smokers, %	18	30	ns
Companionship, %	82	46	0.05
Ethnic origin, %			ns
Caucasian	91	80	-
Oriental	9	17	-
Others	-	Missing	-
Employment, %			0.05
Full time	72	20	-
Part time	1	53	-
Unemployed	18	27	-
Education, %			ns
Primary	-	17	-
Secondary	36	43	-
University	45	33	-
Postgraduate	18	3	-
Family history of			
hyperhidrosis, %	0	43	na

ns = non significant; na = non applicable

**Table 4:** Comparison of correlates of severity of disease in the control and experimental group

	Controls (N=11) mean ± SD	Experimental group (N = 30) mean ± SD	*P*
Global severity	1 ± 0	6.3 ± 1	0.01
Number of topical treatments	0	1.7 ± 1	0.05
Number of medications taken	0	0.8 ± 0.5	NS
Number of showers or baths	1 ± 0.2	2.2 ± 0.6	0.01
Number of clothing changes	1 ± 0.2	2.4 ± 0.5	0.01
Severity of limitations to wardrobe	1 ± 0	5.5 ± 1	0.01
Severity of worsening with alcohol	1.3 ± 0.9	3.6 ± 1	0.01
Severity of worsening with spicy food	2 ± 0.7	3.4 ± 1	0.05
Severity of worsening with coffee	1.4 ± 0.5	2.3 ± 1	0.01

**Table 5:** Comparison of responses to the questions of the Illness Intrusiveness Rating Scale in controls and in the experimental group before and after surgery

	Controls (N =11) mean ± SD	Experimental group before surgery (N = 30) mean ± SD	*P*†	Experimental group after surgery (N = 12) mean ± SD	*P*††
Health	1 ± 0	3.3 ± 7.6	<0.01	1.3 ± 2.4	0.004
Diet	1.1 ± 0.2	3.3 ± 7.3	<0.01	1.4 ± 2.4	0.008
Work	1.1 ± 0.2	6.0 ± 3.5	<0.01	1.8 ± 6.9	0.000
Active recreation	1.3 ± 0.5	5.8 ± 5.5	<0.01	2.2 ± 6.9	0.000
Passive recreation	1 ± 0	3.3 ± 7.6	<0.01	1.3 ± 4.2	0.015
Financial situation	1 ± 0	3.4 ± 5.9	<0.01	1.3 ± 3.1	0.002
Relationship: spouse	1 ± 0	5.0 ± 6.2	<0.01	1.5 ± 4.2	0.000
Sex life	1 ± 0	4.9 ± 8.7	<0.01	1.5 ± 5.9	0.001
Family relations	1 ± 0	3.4 ± 6.9	<0.01	1.3 ± 4.2	0.002
Social relations	1 ± 0	5.9 ± 4.2	<0.01	1.6 ± 5.2	0.000
Self expression	1 ± 0	5.0 ± 5.9	<0.01	1.5 ± 5.9	0.001
Religious expression	1 ± 0	2.2 ± 3.5	<0.01	1.0 ± 0.0	0.002
Community	1 ± 0	3.8 ± 7.6	<0.01	1.5 ± 5.9	0.003
Total IIRS score	13.5 ± 0.7	57 ± 14	<0.001	19.3 ± 15	0.000

† Statistical difference between responses in the control and experimental group before surgery

†† Statistical difference between responses in the experimental group before and after surgery

## IMPACT OF ETS ON QUALITY OF LIFE IN PEOPLE WITH HYPERHIDROSIS

We have previously reported the results of our cohort study of quality of life in individuals with hyperhidrosis, using a pre-post design to study the change in quality of life before and after surgery.[4] Patients completed the questionnaires at two postoperative time points: early, defined as within two months after surgery and late, defined as four months or more after surgery. In addition, postoperative patients answered a single item question reflecting the magnitude of the change in their condition since surgery, rated on a seven-point scale (7 = greatest improvement; 1 = minimal or no improvement). Early postoperative data (1 to 2 months) were available in 12 patients and late postoperative data (4 to 34 months) in 19 patients. The median follow-up time for the early and late questionnaires was 30 days (14 to 64 days) and 370 days (118 to 1013 days), respectively.

**Figure 7:**
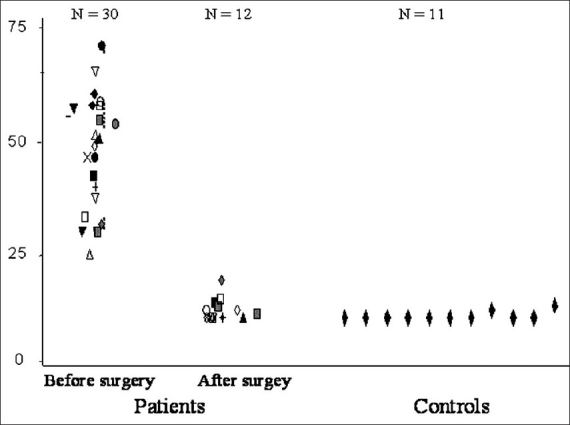
Distribution of the IIRS score before and after early surgery in patients with hyperhidrosis, and in controls

Early after surgery (1 to 2 months) people with hyperhidrosis had a total score which was significantly lower than that before surgery (*P* = < 0.0001) and more in keeping with that of people without hyperhidrosis [Figure 7]. A similar pattern was identified for each of the individual questions of the IIRS. The difference is statistically significant for each of the questions and scale items, for the global severity score and for the total IIRS score at both early and late postoperative time points. For the global severity question, in the preoperative questionnaire the median global severity score was 7 and the lowest response was 5. In the early and late postoperative period the median score was 1, highest 7. For the question on magnitude of change in overall symptoms after ETS, the median score was 7 (lowest 5) at the early post-operative time point and 7 (lowest 2) for the late responses. The improvement in postoperative responses to the IIRS questionnaire was stable as manifested in the late follow up results [Table 6]

**Table 6:** Comparison of preoperative and late postoperative responses to the items of the Illness Intrusiveness Rating Scale for patients with data at both time points

	N	Preoperative mean (SE)	Late postoperative mean (SE)	*P*†
Health	19	4.0 (2.4)	1.0 (0.2)	0.000
Diet	19	3.2 (2.3)	1.1 (0.2)	0.000
Work	19	6.0 (1.3)	1.1 (0.5)	0.000
Active recreation	19	5.9 (1.5)	1.3 (0.8)	0.000
Passive recreation	19	4.0 (2.3)	1.0 (0.2)	0.000
Financial situation	19	3.0 (2.1)	1.0 (0.0)	0.001
Relationship: spouse	19	5.7 (1.3)	1.3 (1.2)	0.000
Sex life	19	5.0 (2.3)	1.1 (0.3)	0.000
Family relations	19	3.6 (2.3)	1.0 (0.0)	0.000
Social relations	19	6.0 (1.5)	1.7 (1.7)	0.000
Self expression	19	5.4 (1.7)	1.2 (0.5)	0.000
Religious expression	19	2.7 (2.1)	1.0 (0.0)	0.003
Community	19	4.0 (2.1)	1.0 (0.0)	0.000
Total	19	58.2 (3.4)	14.9 (1.0)	0.000

† Paired *t* test, comparisons were repeated with Wilcoxon Signed Ranks test and were similar; SE Standard E

The IIRS is an instrument known to be reliable and valid in other chronic conditions[12,13–17,20,21] and we have previously demonstrated its measurement properties in hyperhidrosis, using a different group of patients.[3] Panhofer used two disease-specific questionnaires, devised by Keller *et al.*[6] and Milanez de Campos *et al.*[7] and showed improvement in ratings by both instruments following ETS, adding to the cumulative evidence of effectiveness of ETS and providing evidence for the construct validity of these two instruments. Other cohort studies, using different methods to assess patients’ clinical status, support the effectiveness of ETS in patients with hyperhidrosis, though many of these used unvalidated clinical assessments or questionnaires. A number of retrospective cohort studies,[1,22–24] reported that ETS is safe and effective using an assessment based on a non-quantitative clinical interview at the time of the office visit. Other studies have reported positive outcomes using questionnaires[25–28] or visual analogue scales[29] which appear to have been developed for the purpose of the study and whose reliability and validity were not reported. In addition to using a newly-designed study-specific questionnaire, Sayeed *et al*,[28] reported the results of their prospective cohort study using the Medical Outcomes Study Short Form 36 (SF 36), which has not been validated in the context of hyperhidrosis and was not sensitive to change in their study. Amir validated a disease-specific questionnaire in young Jewish patients with hyperhidrosis.[30] This scale was developed in Hebrew and has not, to our knowledge, been used to measure changes after surgery.

In our study, multiple secondary outcome measures (a global severity item, ten severity questions and a single item rating of improvement with surgery), also gave results which were highly statistically and clinically significant and in keeping with the results of the IIRS. We have also reported statistical comparisons for both early and late postoperative outcomes and our reporting of all outcomes over medium-term follow-up shows that results of surgery were durable up to four years postoperatively. Employment, smoking and maintaining a stable relationship all showed changes in favorable directions after surgery, though these results were not statistically significant. In the study by Ramos and colleagues, anxiety, assessed by the State-Trait Anxiety Index, decreased from 2.08 SD 1.1 preoperatively to 0.39 SD 0.67 postoperatively (*P*<0.001) and the frequency of palpitations, trembling, headache and gastrointestinal disturbance were all also decreased.[19]

A significant weakness of all the available evidence is the use of cohort design where a number of biases are possible: in this setting, patients at the start of a study may magnify the seriousness of their condition with the hope of being considered for treatment and at the end of the study may minimize residual difficulties in order to please the surgeon and staff, the ‘hello-goodbye’ effect.[31] This effect tends to magnify any differences in response to treatment. However, the effects observed in our study[4] were very large and are likely to represent true improvement. Jaeschke *et al*,[32] have suggested that for seven-point Likert scales, changes of 0.5, 1.0 and 1.5 usually correspond to small, medium and large clinical effects, respectively. The changes in the total IIRS score corresponded to a mean decrease of 2 to 3 points in each individual item, which exceeds the differences discussed by Jaeschke by a comfortable margin.

## COMPLICATIONS OF ETS

In our own study, of the 22 patients who underwent surgery, one patient (5%) developed a postoperative pneumothorax requiring placement of a Heimlich valve. There were no other local or wound complications. No patient developed Horner's syndrome or any other neurologic complication. One patient (5%) reported compensatory increase in sweating in the lower lumbar area.

Our findings are in keeping with other studies, which have reported rates of perioperative complications between 4% and 21%.[19,33–36] In a cohort of 281 people undergoing ETS for hyperhidrosis, the rate of Horner's syndrome was 1% and no patient developed gustatory sweating.[37] In 458 patients undergoing ETS reported by Moya and colleagues, major perioperative complications with conversion to thoracotomy occurred in 0.4%, pneumothorax in 2%, subcutaneous emphysema in 1%, pleural bleeding in 0.2%, hemothorax in 0.1% and atelectasis in 0.1%.[38] In this study, excessive dryness of the hands occurred in 0.4%, Horner's syndrome in 0.3% and gustatory hyperhidrosis in 1.1% of patients.

Rates of compensatory hyperhidrosis in our study were low, occurring in one out of 22 patients (5%). This patient had noted increased sweating in the lumbar region since surgery, but her overall scores reflected marked improvement and the patient expressed satisfaction with the procedure. Other studies have reported higher rates of compensatory hyperhidrosis, between 17% and 100%.[34–37,39] Some authors suggest that a more extensive of sympathectomy (to the level of T4 or T5) is associated with increased compensatory hyperhidrosis.[40,41] Leseche *et al.*[42] using multivariate analysis in a cohort of 134 patients undergoing ETS for hyperhidrosis reported an incidence of compensatory sweating of 71%. This was described as minor in 53% and severe in 19%. They found that the extent of sympathectomy was not associated with the occurrence of this complication. In their cohort, however, only 15% of patients had a sympathectomy extending to T3, 48% received a sympathectomy extending to T4 and 37% to T5. In 1,274 patients undergoing ETS reported by Reisfeld, some degree of compensatory sweating was reported in almost all patients, but was usually mild.[41] In this cohort, 2% of patients requested reversal of their procedure.

An advantage of using the IIRS, the Keller or the Milanez de Campos instruments in the study of response to surgery is that complications of surgery and adverse effects such as hyperhidrosis, if they are sufficiently severe that they impact upon the patient's life, will be reflected in a more severe total score. With respect to compensatory hyperhidrosis, we believe that the frequency and severity of this complication may be reduced by limiting the sympathectomy to T2–3 in most patients and reserving more extensive sympathectomy (T2–4 or T2–5) only to those with hyperhidrosis limited to the axillae or after failure of a previous sympathectomy. Our study suggests that whatever the true incidence of this complication is, patients feel that overall surgery improved their life in the large majority of cases. A thorough informed consent however is necessary to explain the nature and incidence of complications after ETS.

## SELECTION OF PATIENTS FOR ETS

As the high preoperative scores in our study show, patients referred to surgical practices suffered from severe manifestations of hyperhidrosis.[4] Given the possibility of complications from surgery, we believe that surgery should be reserved for those with the most severe manifestations and after other less invasive options have been exhausted.

## FUTURE DIRECTIONS

Surgeons should continue to report the results of cohort studies of ETS, including long-term results. A randomized controlled trial in people with severe hyperhidrosis, comparing ETS with continued best medical management or with repeated botulinum toxin injections, would be a valuable addition to the literature.

## SUMMARY AND CONCLUSIONS

Severe hyperhidrosis is severely intrusive and greatly affects people's lives. In cohort studies with medium-term follow-up, surgery appears to be safe and effective for the treatment of this condition. Given the possibility of complications, we believe that surgery should be reserved for patients with severe hyperhidrosis. In clinical practice, severity can be usefully defined by the use of a self-administered questionnaire containing the IIRS and the ten severity questions, the Keller questionnaire or the Milanez de Campos questionnaire and these same instruments can be used to monitor the response to treatment.
